# Non-Isothermal Crystallization Kinetics of Poly (ɛ-Caprolactone) (PCL) and MgO Incorporated PCL Nanofibers

**DOI:** 10.3390/polym15143013

**Published:** 2023-07-12

**Authors:** Daisaku Gicheha, Aicha Noura Cisse, Ariful Bhuiyan, Nabila Shamim

**Affiliations:** 1 Department of Chemical Engineering, Prairie View A & M University, Prairie View, TX 77446, USA; dgicheha@pvamu.edu (D.G.); acisse1@pvamu.edu (A.N.C.); 2 Mechanical Engineering Program, University of Houston Clear Lake, Houston, TX 77058, USA; bhuiyan@uhcl.edu

**Keywords:** nanofibers, electrospinning, non-isothermal crystallization

## Abstract

The study delves into the kinetics of non-isothermal crystallization of Poly (ɛ-caprolactone) (PCL) and MgO-incorporated PCL nanofibers with varying cooling rates. Differential Scanning Calorimetry (DSC-3) was used to acquire crystallization information and investigate the kinetics behavior of the two types of nanofibers under different cooling rates ranging from 0.5–5 K/min. The results show that the crystallization rate decreases at higher crystallization temperatures. Furthermore, the parameters of non-isothermal crystallization kinetics were investigated via several mathematical models, including Jeziorny and Mo’s models. Mo’s approach was suitable to describe the nanofibers’ overall non-isothermal crystallization process. In addition, the Kissinger and Friedman methods were used to calculate the activation energy of bulk-PCL, PCL, and MgO-PCL nanofibers. The result showed that the activation energy of bulk-PCL was comparatively lower than that of nanofibers. The investigation of the kinetics of crystallization plays a crucial role in optimizing manufacturing processes and enhancing the overall performance of nanofibers.

## 1. Introduction

Polymer nanofibers are gaining significant attention due to their potential for developing materials with tailored properties for various applications, including but not limited to oral drug delivery [1], wound healing [2], fine particle filtration [3], tissue engineering [4], optoelectronics [5], and sensor technology [6]. The efficacy of nanofibers in scaffold fabrication is contingent upon their surface-to-volume ratio, the porosity of the nanofiber mesh, and distinctive physicochemical characteristics [7]. Electrospinning has been the most prevalent technique for producing nanofibers. Due to the rapid stretching of the electrical jet and evaporation of the solvent during the electrospinning process, a portion of the polymer remains non-crystalline. The non-crystalline polymer chain eventually becomes entrapped between the growing crystals [8]. Recent research by Soleimani et al. [9] on the structure-property relationship of randomly aligned polylactide revealed that spun fibers consist of crystalline and mesomorphic phases as well as oriented but mobile amorphous chain segments. According to Dimitry et al. [10], the mechanical properties significantly influences the practical applications of nanofibers. These properties can be modified by manipulating their internal structure, which includes their nanoscopic and substructural characteristics. The microstructure and characteristics of fibers and the scaffold they form are directly influenced by the molecular alignment of nanofibers produced through electrospinning. The size of electrospun fibers is limited by the interaction between electrical and mechanical forces that cause polymer chains to align [11]. However, several aspects of the relationship between structure and property still require further investigation. Therefore, it is crucial to comprehensively examine the internal structure of polymeric nanofibers to enhance their efficiency, a domain that has yet to be extensively explored.

Thermal analysis is a technique used to examine changes in the structural composition of semicrystalline polymer fibers and assess the influence of fiber conformation on mobility. Recently, Xu et al. [12] reported the existence of cylindrical structures in electrospun polycarbonate fibers, which resemble the super molecular structure initially postulated by Arinstein et al. [13]. It has been reported that during the process of crystallization, non-crystalline polymer chains may become entrapped. These chains are embedded between the growing crystals either within or outside the lamella structure. In addition, Ma et al. [8] evaluated the confinement effect in aligned Poly (lactic acid) (PLA) nanofibers and compared the rigid amorphous phase with the mesophase. According to their report, the devitrification of the mesophase serves as the primary driving force for crystallization, resulting in an acceleration of the process at a lower temperature compared to unoriented PLA film. The process of crystal formation is significantly impacted by the phenomenon of nanoconfinement, resulting in modifications to the crystallization behavior. Therefore, the interpretation of the non-crystalline phase of electrospun fibers is of interest and can enhance the phase confinement effect in nanofibers. In addition, such research necessitates approaches to molecular orientation and molecular simulation to characterize the freeze-in stress that influences the nucleation mechanism and crystalline structure. This research examines the non-isothermal crystallization kinetics of randomly aligned polycaprolactone nanofibers in this context. In a non-isothermal crystallization process, the temperature of crystallization varies with time. Due to the complexity of the non-isothermal crystallization method, various modified Avrami models [14,15,16], such as the Jeziorny model [17], Ozawa’s model [18], and Lui-Mo’s model [17,18,19] have been developed to comprehend the process better. Previously, these models were utilized frequently to comprehend bulk polymeric materials’ non-isothermal crystallization kinetics behavior [20,21,22,23].

Poly (ɛ-caprolactone) (PCL) is a type of aliphatic, bio-degradable polyester commonly utilized for implantable biomaterials due to its non-toxic and crystallizable nature. The non-toxic and biodegradable properties of PCL are suitable for various applications, including medical, pharmaceutical, and tissue engineering, and drug delivery systems [24]. The material’s lack of toxicity and ability to degrade naturally render it a viable option for utilization in tissue engineering. The biodegradability of a solid polymer is influenced by its chemical structure. As per the findings, the degradation rate of PCL is primarily influenced by the crystallinity feature of its semicrystalline shape [25]. It is a semicrystalline polymer with a low melting point of 59–64 °C and a glass-transition temperature of −60 °C, and it remains in a rubbery state at room temperature [26]. The literature demonstrates that molecule orientation influences the viscoelastic characteristics, degree of crystallinity, and crystal size of electrospun mats [27].

Tailored composite nanofibers have exhibited significant potential for biomedical applications in addition to polymeric nanofibers. Magnesium (Mg) is essential for the optimal functioning of nerve tissue and recovery from nerve damage, as indicated by previous studies [28,29,30]. The selection of MgO in this research was based on its advantageous biodegradability and biocompatibility properties. Research findings indicate that increasing the degree of crystallinity in polycaprolactone (PCL) can extend the duration of degradation and drug release kinetics [31,32]. According to the literature [33], MgO can be incorporated into a polymer to customize its properties for drug delivery purposes. Although progress has been made in producing nanofiber membranes that incorporate MgO, a more thorough understanding of the thermal characteristics of these composites is still required. The objective of our study is to analyze the thermal characteristics of a group of randomly arranged PCL and MgO-PCL nanofiber mats as opposed to individual nanofibers. A collection of nanofiber mats replicates the actual application conditions and is relatively easier to manipulate than a single fiber. Magnesium was selected based on its outstanding biocompatibility and capacity to biodegrade into non-toxic compounds.

The extent of crystallinity in semi-crystalline polymeric scaffolds has been observed to impact various properties, such as surface free energy, wettability, solubility, and degradation behavior [34,35]. Nanofibers are generated through rapid evaporation, which results in the polymer chains adopting energetically unfavorable orientations. The crystals formed under these particular conditions exhibit a conformation with high energy [8]. However, the extent of thermal treatment can impact the level of crystallinity, chain relaxation, and decrease in chain energy. Furthermore, there has been a notable surge of interest in the crystallization of polymers within confined spaces, as nano-confinement can impede crystal growth and lead to significant deviations in crystallization behavior from that observed in bulk conditions [8,13]. Therefore, understanding the crystal kinetics of polymer nanofibers require fundamental knowledge in crystallization process to control the structures and properties of the final scaffold. This study aims to conduct a thorough investigation of the non-isothermal crystallization of PCL and MgO-PCL nanofibers and draw a comparison with the bulk material by utilizing Differential Scanning Calorimetry. The dynamics of non-isothermal crystallization have been explored using Mo’s model to comprehend the complex non-isothermal crystallization. The activation energy of confined polymer in electrospun nanofibers was then calculated using the Kissinger and Friedman method.

## 2. Experimental

### 2.1. Materials and Methods

The electrospinning technique was employed to fabricate the nanofibers, following the methodology outlined in a prior study [36]. The concentration of Poly (ɛ-caprolactone) from Scientific Polymer Products (Mw 70,000 estimated by gel permeation chromatology {GPC}, CAS No # 24980-41-4, Ontario, NY, USA) in the solution was 10 wt.% and 5 wt.% magnesium oxide was incorporated in Poly (ɛ-caprolactone) solution to fabricate MgO-PCL nanofiber composites. Commercial MgO from Sigma Aldrich (CAS No# 1309-48-4, St. Louis, MO, USA) with a particle size less than 50 nm was used in the study. The drum collector was spun using a direct current (DC) motor. By applying a high voltage (10 kV) generated by the Gamma High Voltage power source, the syringe needle was electrically energized. This electrically-charged syringe needle was positioned above a drum collector to capture the PCL-aligned fiber stream. The distance between the needle and drum collectors was approximately 5 cm. The feeding rate of the PCL solution was adjusted to a rate of 0.025 mL/min. Poly (ɛ-caprolactone) cloths were directly collected on a drum with a 2-inch diameter. We optimized the rotation speed, the distance between the needle and drum, and the fiber deposition rate onto the drum. A sterilized sharp razor blade was used to cut the cloth into dimensions 18 cm long and 16 cm wide from the drum to be used for experimentation. The chemical composition and processing conditions are shown in Table 1. Bulk-PCL was prepared by mixing 10 wt.% of Poly (ɛ-caprolactone) in Acetone by using sonication. Once blended, PCL solution was dried inside a low-heat vacuum oven.

### 2.2. Differential Scanning Calorimetry

**Figure 1:**
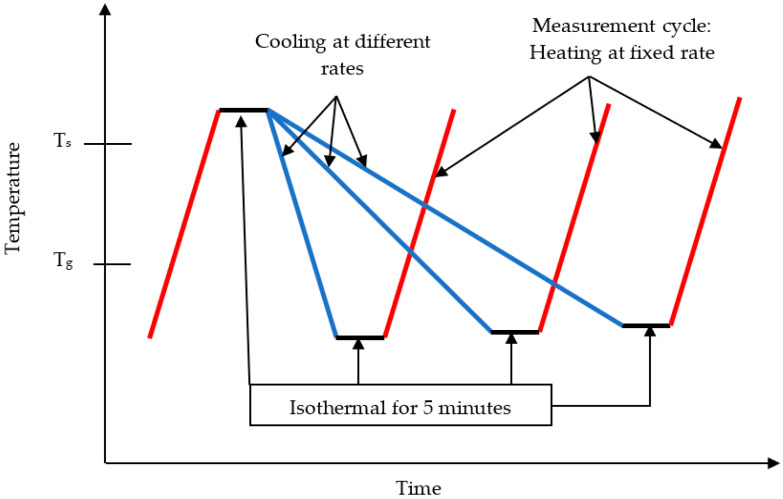
A schematic diagram of the experimental method used to investigate the non-isothermal crystallization of Bulk-PCL, PCL, and MgO-PCL nanofibers.

Differential scanning calorimetry (DSC-3) from METTLER TOLEDO (Columbus, OH, USA) was utilized to conduct non-isothermal crystallization kinetic investigations. The Star-E system software from METTLER TOLEDO was utilized to assess the outcomes of the DSC analysis. The experiments are conducted under a nitrogen atmosphere at a 50 mL/min flowrate. Calibration was executed utilizing high purity indium in order to guarantee the precision of the outcomes. The specimens were measured in weight and subsequently positioned within conventional aluminum crucible pans with a volume of 40 µL. The experimental procedure was carried out in a series of concurrent runs, as shown in a schematic diagram in Figure 1. The samples were heated from 25 °C to 90 °C at 10 K/min, and held at 90 °C for five minutes to remove any thermal history. Then, the samples were cooled at different cooling rates ranging between 0.5–5 K/min.

The nanofibers were cut into thin strips along the fiber axis for DSC tests. The small fiber mats were then freely placed on the aluminum pan, and the lid was then closed by gentle mechanical compression. It will allow the most-free relaxation of the polymer chains [8]. The kinetics behavior was analyzed by using the METTLER TOLEDO STARe software. Peak curve integration was used to measure the reaction enthalpy for each reaction. The STARe software includes a conversion function that generates conversion curves and tables. The generated curves are exported to Excel, enabling thorough conversion curves analysis.

## 3. Results & Discussions

### 3.1. Non-Isothermal Crystallization Properties

**Figure 2:**
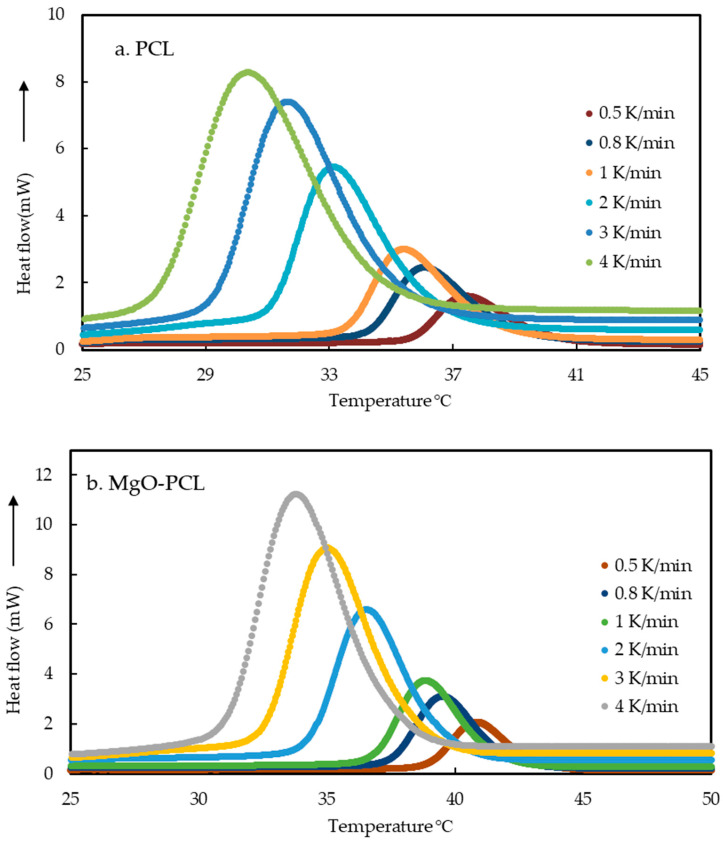

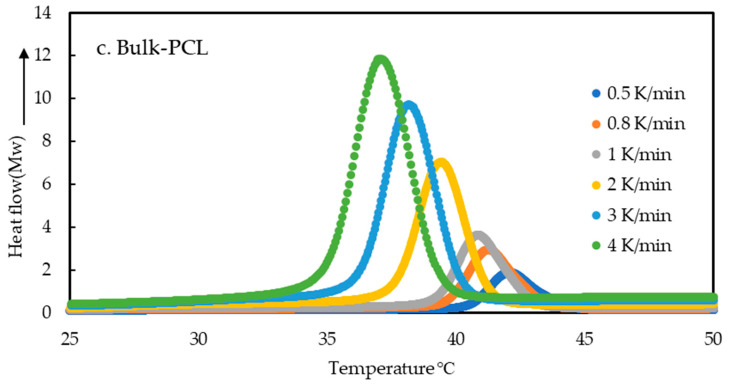
(**a**–**c**). Non-isothermal crystallization exotherms of (**a**) PCL, (**b**) MgO-PCL, and (**c**) bulk PCL measured at various cooling rates ranging of 0.5–4 K/min.

The cooling cycles of DSC curves for confined PCL, MgO-PCL nanofibers, and bulk-PCL are shown in Figure 2a–c. The exothermic peak observed is attributed to its crystallization temperatures and depends on the cooling rates. Experiments were conducted at a higher cooling rate of 10–30 K/min, but no crystallization peaks were detected. The values of (*T_o_*) and (*T_p_*) can be utilized to ascertain the temperature at which the crystallization process commences and the highest temperature of the crystallization peak, respectively. A shift toward higher crystallization temperature was noted with a decreased cooling rate. The data suggest that an increase in cooling rates results in a reduction of the crystallization temperature. In summary, a direct relationship exists between the rate of cooling and the extent of supercooling [31,32,37]. According to Xu et al. [38], when cooling rates are high, the crystallization time available to the crystalline entities is reduced, leading to the inability of the nuclei in the molten matrix to crystallize. Consequently, a disordered solid is formed. Furthermore, it has been postulated [39] that molecular chains at high cooling rates have less time to diffuse into the crystallite lattice, modify and organize their configurations, and form more perfect crystallites. Table 2 summarizes the crystallization behavior of both bulk and confined nanofibers under different cooling rates. The results indicate that the PCL nanofiber crystallization temperature (37.54 °C ± 0.22) is lower than that of MgO-PCL (41.17 °C ± 0.37) at a cooling rate of 0.5 k/min. The observed phenomenon was ascribed to the nucleation capacity of the fibers containing MgO nanoparticles, which facilitate the polymer matrix’s crystallization process, particularly in the interface area between MgO-PCL in the composite materials [32,40]. As a result, such a process should occur at temperatures, increasing both *T_p_* and *T_o_*.

Table 2 presents the data on the characteristics of non-isothermal crystallization exotherms for bulk-PCL, PCL, and MgO-PCL nanofibers. The variable *T_p_* denotes the temperature at which the maximum rate of crystallization occurs, while *t*_0.5_ refers to the duration of time required for half of the crystallization process to take place. Additionally, *t*^−1^_0.5_ represents the reciprocal of the crystallization half-time. The phenomenon of crystallization can be elucidated in the following manner. The amorphous polymer matrix of pure polymer contains a small number of impurities in the form of unreacted monomers dispersed randomly throughout the domains. Consequently, the formation of pure domains will occur through homogeneous crystallization, requiring a significantly reduced temperature for nucleation. On the contrary, it has been observed that PCL nanofibers incorporating MgO exhibit heterogeneous crystallization at comparably elevated temperatures. Comparable outcomes were noted for polyethylene oxide (PEO) confined within polystyrene (PS) nanofibers having a vitreous exterior [19].

The relative crystallinity as a function of the cooling rate was obtained from the DSC curves. During the crystallization process at a constant cooling rate (*β*), the relative degree of crystallization *X_t_* within the matrix of confined PCL, MgO-PCL nanofiber, and bulk-PCL materials after a given crystallization time, *t*, can be determined using Equation (1), as follows [41]:

(1) $$X_{t}=\frac{\int_{T_{o}}^{T}\left(dH_{c}/dT\right)dT}{\left(1-\psi \right)\Delta H_{c}}$$

where *dH_c_* is the enthalpy of crystallization during the time interval *dT*, *T_o_* is the onset temperature, *T* arbitrary temperature at time *t*, (1 − *ψ*) signifies the weight fraction of the polymers, and Δ*H_c_* corresponds overall enthalpy of 100% crystalline PCL that has been previously determined to be 139 J/g [41]. Typically, the achievement of relative crystallization involves the integration of the region beneath the exothermic peak in the heat flow curves. Furthermore, under non-isothermal circumstances, it is possible to transform the crystallization temperatures into crystallization time [14], marked as Equation (2):

(2) $$t=\left|T-T_{O}\right|/\beta$$

where β is the cooling rate applied for the non-isothermal crystallization. The non-isothermal crystallization exotherms of PCL, MgO-PCL nanofibers, and bulk-PCL obtained are shown in Figure 2a–c. Crystallization kinetics are widely analyzed by the Avrami equation [15,16]. The overall effect of the cooling rate of bulk-PCL, PCL, and MgO-PCL nanofiber was investigated to determine the overall crystallization time (*t_c_*). The following equation, Equation (3), was used to calculate each *t_c_* of the samples:

(3) $$t_{c}=\frac{T_{o}-T_{end}}{\beta }$$

where *T_o_* is the onset crystallization temperature, *T_end_* is the finishing crystallization temperature, and *β* is the cooling rate considered. Table 2 demonstrates a significant reduction in crystallization time with an increased cooling rate. The above outcome is expected due to the accelerated cooling rate impeding the development of crystalline structures within the molten matrix, consequently diminishing the duration of the materials’ crystallization process. Furthermore, it can be observed from Table 2 that the composite nanofiber exhibits a reduced crystallization time (*t_c_*) in comparison to the pure PCL nanofiber. The acceleration of the crystallization process in MgO-incorporated PCL and the consequent reduction in crystallization time can be attributed to the inclusion of heterogeneities. In comparison to confined PCL and MgO-PCL nanofibers, non-electrospun bulk-PCL exhibits the shortest crystallization time. According to Coburn et al. [42], the bulk-PCL exhibits a relative crystallinity of 60% when subjected to a 2 K/min cooling rate. The level of crystallinity exhibits a reduction in the case of confined nanofibers. The restricted mobility of polymer chains within smaller domains is a probable cause for the hindered development of crystallinity. 

#### 3.1.1. Kinetics of Non-Isothermal Crystallization

**Figure 3:**
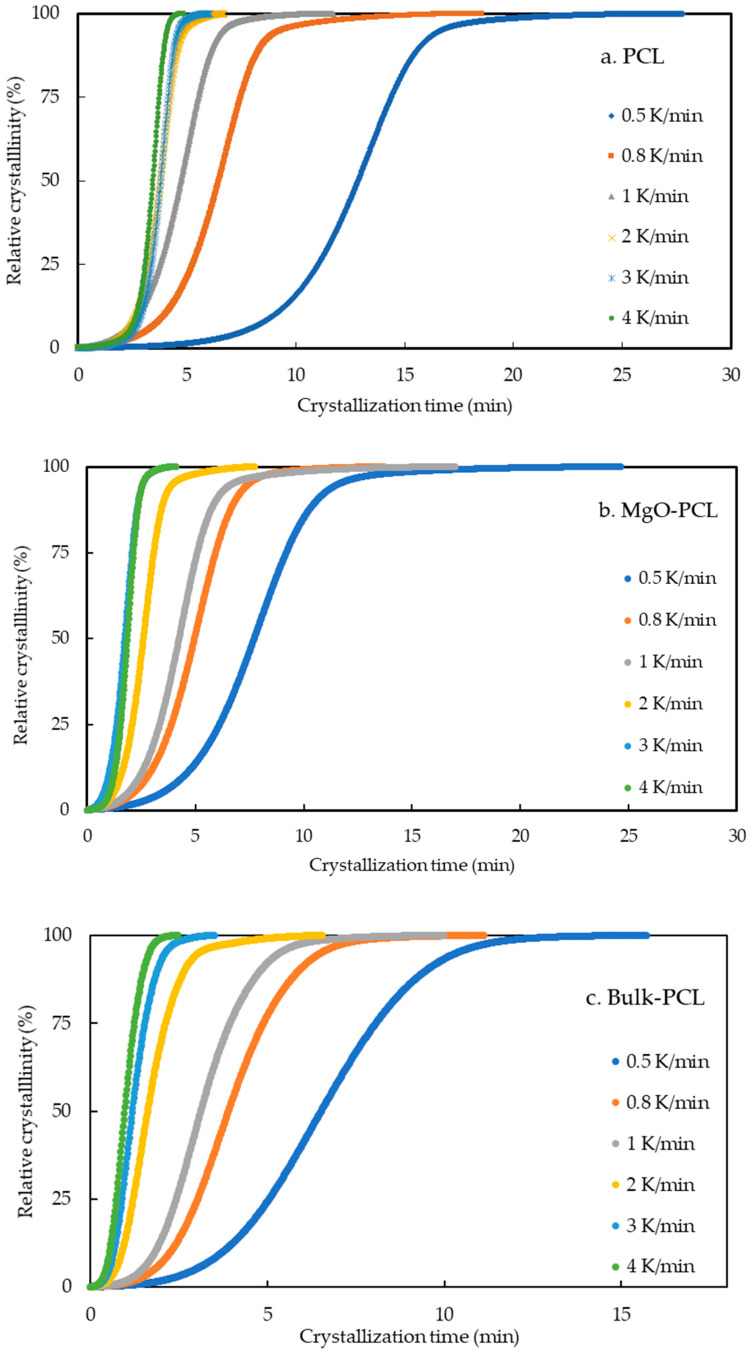
(**a**–**c**). Plot of relative crystallinity verses crystallization time for the (**a**) PCL, (**b**) MgO-PCL and, (**c**) bulk-PCL at various cooling rates.

The relative crystallinity versus crystallization time profiles for bulk-PCL, PCL, and MgO-PCL nanofibers samples at various cooling rates are depicted in Figure 3a–c. Each curve demonstrates a time dependence that follows a sigmoidal pattern. The leftward shift of sigmoid curves was observed with increased cooling rates. This suggests that an increase in the cooling rate reduces the time needed to achieve maximum crystallinity. Consequently, an increase in the cooling rate leads to a decrease in the half-life *t*_0.5_ values, as evidenced by the DSC curves. In the context of crystallization, the relative degree of crystallization *X_t_* can be ascertained for confined PCL, MgO-PCL nanofiber, and bulk-PCL materials by subjecting them to a constant cooling rate (*β*) during the crystallization process and measuring the resulting *X_t_* after a given crystallization time *t*. This can be achieved through the application of the following methodology.

The rate of crystallization is defined as the inverse time it takes for crystallization to reach 50% is expressed as:

(4) Rate t0.5−1=1t0.5

**Figure 4:**
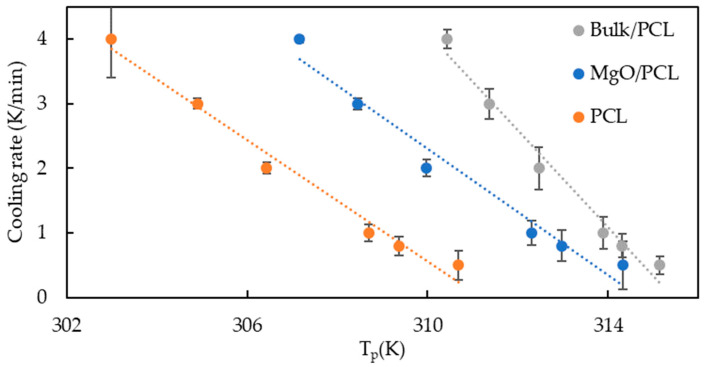
Plot of cooling rate as a function of the temperature at the maximum crystallization rate (CRC). Error bars represent standard deviation from three measurements on each sample.

**Figure 5:**
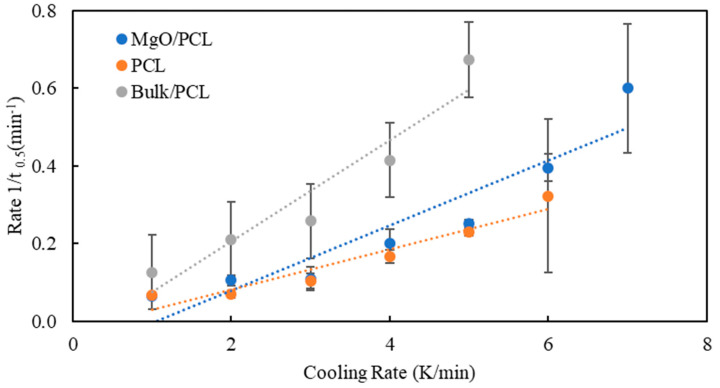
Plot of reciprocal half-time of crystallization as a function of the heating rate of the bulk-PCL, PCL, and MgO-PCL nanofibers (CRP). Error bars represent standard deviation from three measurements on each sample.

The non-isothermal crystallization rates are compared through the crystallization rate coefficient (CRC) and crystallization rate parameter (CRP). According to crystallization half time, crystallization rate co-efficient (CRC) and crystallization rate parameter (CRP) evaluate the crystallization rate. CRC defines the variation in cooling rate to change the undercooling of the polymer melt by 1 °C. CRC categorizes polymers according to their slope. A direct relationship exists between the crystallization rate and the slope’s steepness. The slope obtained by CRP ascertains the relative position of the crystallization rate. Figure 4 illustrates, through the utilization of the CRC ranking methodology, that the incline of bulk samples is more pronounced than that of nanofibers. The examination of the crystallization rate of nanofibers and bulk PCL indicates that the application of nano-confinement results in a reduction of the crystallization rate. This is demonstrated by the data presented in Figure 4 and Figure 5:

The data presented in Table 2 indicate that the peak temperature (*T_p_*) in degrees Celsius and the half-time of non-isothermal crystallization (*t*_0.5_) in minutes decrease as the cooling rate increases. The observed reduction in *t*_0.5_ values suggests an acceleration in the crystallization process with an increased cooling rate. It has been observed that there is a positive correlation between the cooling rate and the *t*^−1^_0.5_ values, indicating that the latter tends to increase as the former increases. This observation suggests that there exists a positive correlation between the cooling rate and the rate of crystallization, whereby an increase in the former leads to a corresponding increase in the latter.

#### 3.1.2. Non-Isothermal Mathematical Modeling

Numerous mathematical models have been developed to depict non-isothermal mathematical modeling. The investigated models in this study include the Jeziorny-modified Avrami equation, Ozawa equation, Case Average Avrami exponent, Chuah Average Avrami exponent, and Combined Avrami/Ozawa Equation, as well as Kissinger and Friedman activation energy.

##### Jeziorny Modified Avrami Equation

A widely used Avrami model [17] for describing isothermal crystallization kinetics for polymers, as shown in Equation (5), is formulated as:

(5) $$1-X_{t}=exp\left(-Z_{t}t^{n}\right)$$

where *Z_t_* is Avrami crystallization rate constant and *n* is the Avrami exponent. *Z_t_* and *n* parameters can be calculated by transforming Equation (5) into a traditional linear form Equation (6):

(6) $$\log\;\left\{-\ln\left[1-X_{t}\right]\right\}=n\;\log\;t+\log Z_{t}$$

where a linear relationship can be seen when plotted $\log\;\left\{-\ln\left[1-X_{t}\right]\right\}$ vs. log *t*.

The Jeziorny model postulates that the crystallization temperature remains constant and adjusts the Avrami parameters to investigate the kinetics of non-isothermal crystallization in polymers. This is achieved by assuming that the rate of cooling remains constant. The recalibrated rate constant for crystallization is presented as follows:

(7) $$\log Z_{c}=\log Z_{t}/\beta$$

This equation is commonly used in academic literature to describe the non-isothermal crystallization kinetics of polymers, where *Z_c_* is a parameter utilized for this purpose, and where *Z_C_* parameter can describe the non-isothermal crystallization kinetics for polymers and *β* is the cooling rate. The determination of the Jeziorny parameters *Z_c_*, *Z_t_*, and Avrami exponent, *n*, can be achieved by plotting the logarithm of $\log\;\left\{-\ln\left[1-X_{t}\right]\right\}$ against log *t* using the following Equations (6) and (7). The *n* and *t* values were determined from the slope and intercept of the linear plots.

**Figure 6:**
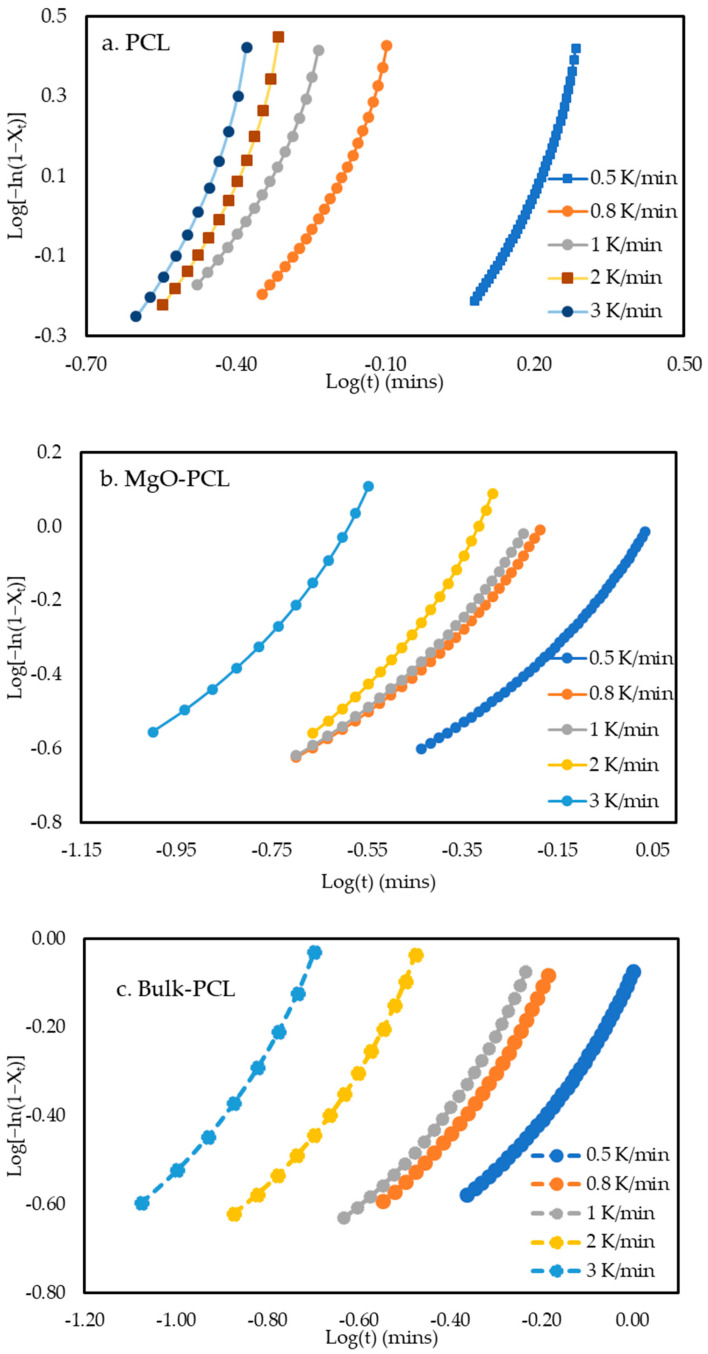
(**a**–**c**). Plot of log {−ln[1 − *X_t_*]} against log of (**a**) PCL, (**b**) MgO-PCL and (**c**) Bulk-PCL according to Jeziorny model at various cooling rates.

Jeziorny corresponding plots of bulk-PCL, PCL, and MgO-PCL nanofibers are shown in Figure 6a–c. The primary crystallization stage shows a good linear relationship for all samples. The linear part was used to calculate *n*, *Z_t_*, and *Z_c_* values, and are listed in Table 3. Pearson’s correlation coefficient *R*^2^ for all samples are close to 1, and support the linearity of the plots. The values of *n* exhibited variability ranging from 1.2 to 1.4 across bulk-PCL, PCL, and MgO-PCL nanofibers under different cooling rates. The values denoted by *n* signify a growth phenomenon in a single dimension [17]. The results indicate a positive correlation between cooling rate and *Z_t_* values, as the average *Z_t_* values tend to increase with an increase in cooling rate.

##### Mo’s Method

Mo and Liu [43,44] proposed a new approach to model the kinetics of non-isothermal crystallization by integrating the Avrami equation and Ozawa equation [45]. Equation (9) is calculated by combining Equations (6) and (8), as described below:

(8) $$\ln\;\left\{-\ln\left[1-X_{t}\right]\right\}=\ln K\left(T\right)-m\;\ln\beta$$

(9) $$\log Z_{t}+n\log t=\log K\left(T\right)-m\log\beta$$

By solving log *β*, we get:

(10) $$\log\beta =\frac{1}{m}\log\left[\frac{K\left(T\right)}{Z_{t}}\right]-\frac{n}{m}\log t$$

**Figure 7:**
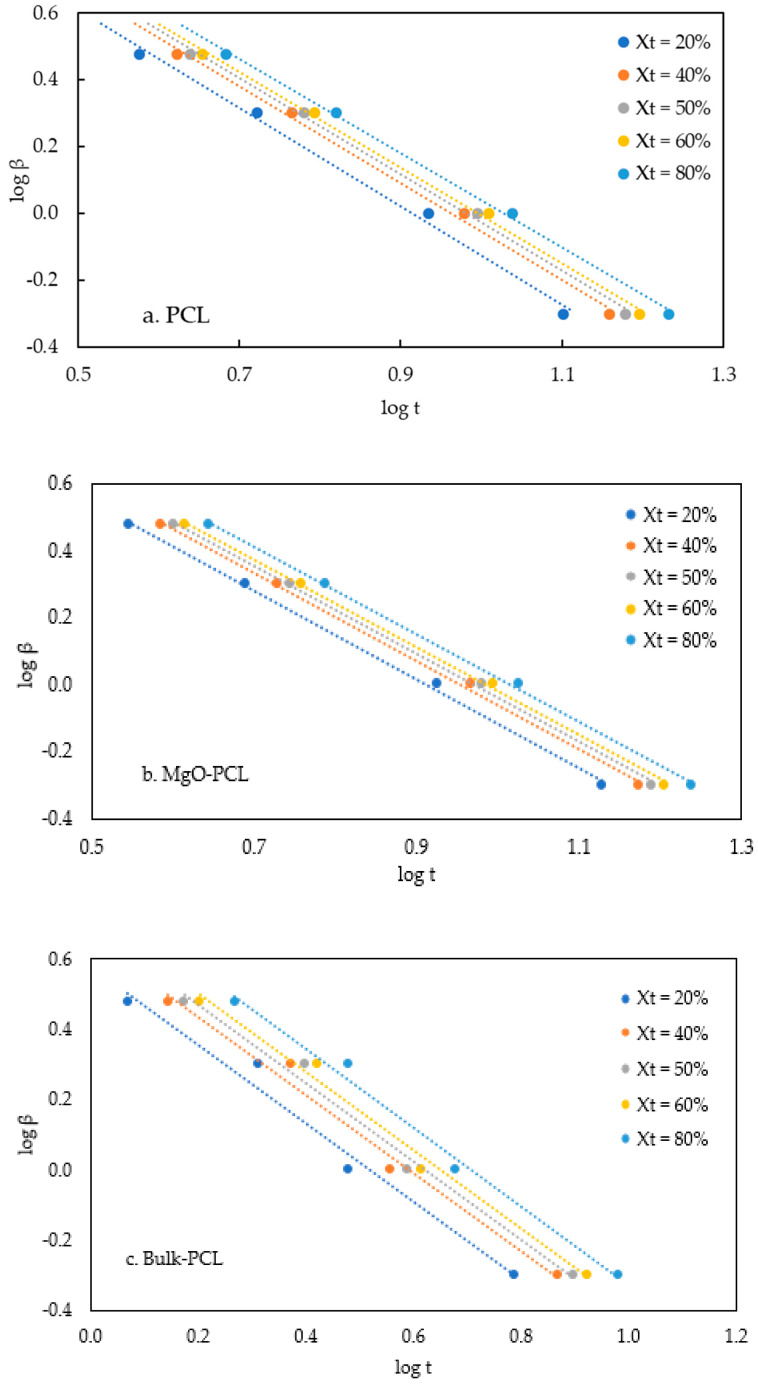
(**a**–**c**). Plot of log *β* verses log *t* from Mo’s method for non-isothermal crystallization for (**a**) PCL, (**b**) MgO-PCL, and (**c**) bulk-PCL. In this figure, *β* is cooling rate in K/min and *t* is time in minutes.

Let $F\left(T\right)={\left[\frac{K\left(T\right)}{Z_{t}}\right]}^{1/m}$ and $\alpha =\frac{n}{m}$;

The final equation is transformed to:

(11) $$\log\beta =\log F\left(T\right)-\alpha \log t$$

where *F*(*T*) is the cooling value chosen at a crystallization time when the system has a certain degree of crystallinity and *α* is the ratio of Avrami exponent and Ozawa exponent. Avrami exponent (*n*) and Ozawa exponent (*m*) hinge on the type of nucleation and growth mechanism. Figure 7a–c depict the log *β* versus log *t* variations for specific relative degrees of crystallinity *X_t_*, namely 20%, 40%, 50%, 60%, and 80%, in both pure and composite nanofibers, as examined in this investigation. The values of *F*(*T*) and *α* can be derived by utilizing the intercept and slope.

The results show that the higher the cooling rate, the shorter the crystallization time for a specific relative crystallinity. The linearity as shown from these plots best describes Mo’s equation.

The correlation between *α*, *F*(*T*) and the rate of crystallization is presented in Table 4. The slope (*α*) values for Bulk-PCL, PCL, and MgO-PCL nanofibers remain constant at 1.1, 1.4, and 1.3, respectively. The observed values of *α* indicate that the crystallization structures formed are highly similar, despite variations in relative crystallinities. Furthermore, the slight variance observed in the *α* values of the bulk-PCL, PCL, and MgO-PCL nanofibers suggests that the approach employed by Mo et al. (as described in Equation (12)) effectively elucidates the non-isothermal process associated with PP-clay nanocomposites [46] and PP-surface-treated SiO_2_ nanocomposites [47].

As the relative crystallinity increases, there is a corresponding increase in the value of *F*(*T*). A greater cooling rate is necessary within a designated crystallization period to achieve increased crystallinity. The *F*(*T*) value associated with PCL nanofibers is comparatively more significant than that of MgO-PCL nanofibers. The findings indicate that attaining a particular relative crystallinity level necessitates a lower cooling rate for MgO-PCL nanofibers compared to MgO-PCL nanofibers. At a specific degree of crystallization (*X_t_* = 20%), it has been observed that the *F*(*T*) value of MgO-PCL nanofibers is comparatively lower than that of PCL nanofibers. Adding a nucleating agent to PCL nanofibers results in an accelerated crystallization rate, as evidenced by the data. The crystallization rate of a polymer in a bulk state (bulk-PCL) is comparatively higher than that of polymers in a confined state (PCL and MgO-PCL nanofibers).

### 3.2. Kissinger Activation Energy

Two distinct factors govern the process of polymer crystallization. The first factor is dynamic and pertains to the activation energy Δ*E* required for transporting crystalline units across the phase. The second factor is static and is associated with the free energy barrier for nucleation [48]. The Kissinger method is commonly employed for determining the activation energy associated with non-isothermal crystallization, as investigated through differential scanning calorimetry (DSC). The Kissinger equation is expressed in the following manner [49]:

(12) $$\frac{d\left[\ln\left(\frac{\beta }{T_{p}{}^{2}}\right)\right]}{d\left(\frac{1}{T_{p}}\right)}=-\frac{\Delta E}{R}$$

where β is the cooling rate (K/min), *R* is the gas constant, and *T_p_* is the peak temperature at its maximum value.

**Figure 8:**
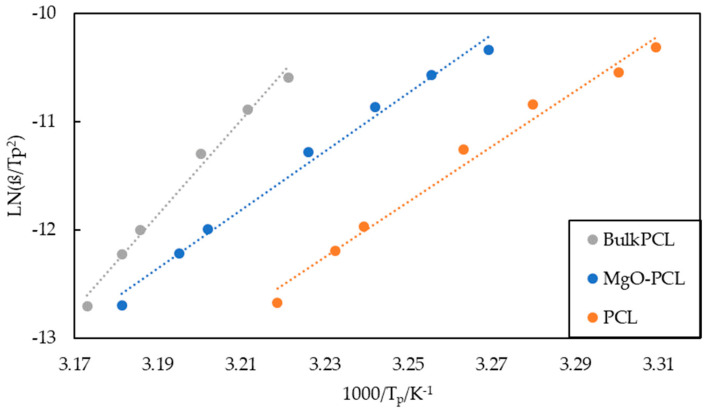
Kissinger’s plot for bulk-PCL, PCL and MgO-PCL nanofibers.

The Δ*E* can be estimated through the Kissinger method by utilizing Equation (12) and determining the slope of a linear plot of ln (β/*T_p_*^2^) versus 1/*T_p_*, as shown in Figure 8 and Table 5. According to Tianxi Liu et al. [50], the activation energy, denoted as Δ*E*, encompasses both the transport activation energy necessary for the transportation of molecular segments across the phase boundary and the energy required for crystallization on the surface.

**Figure 9:**
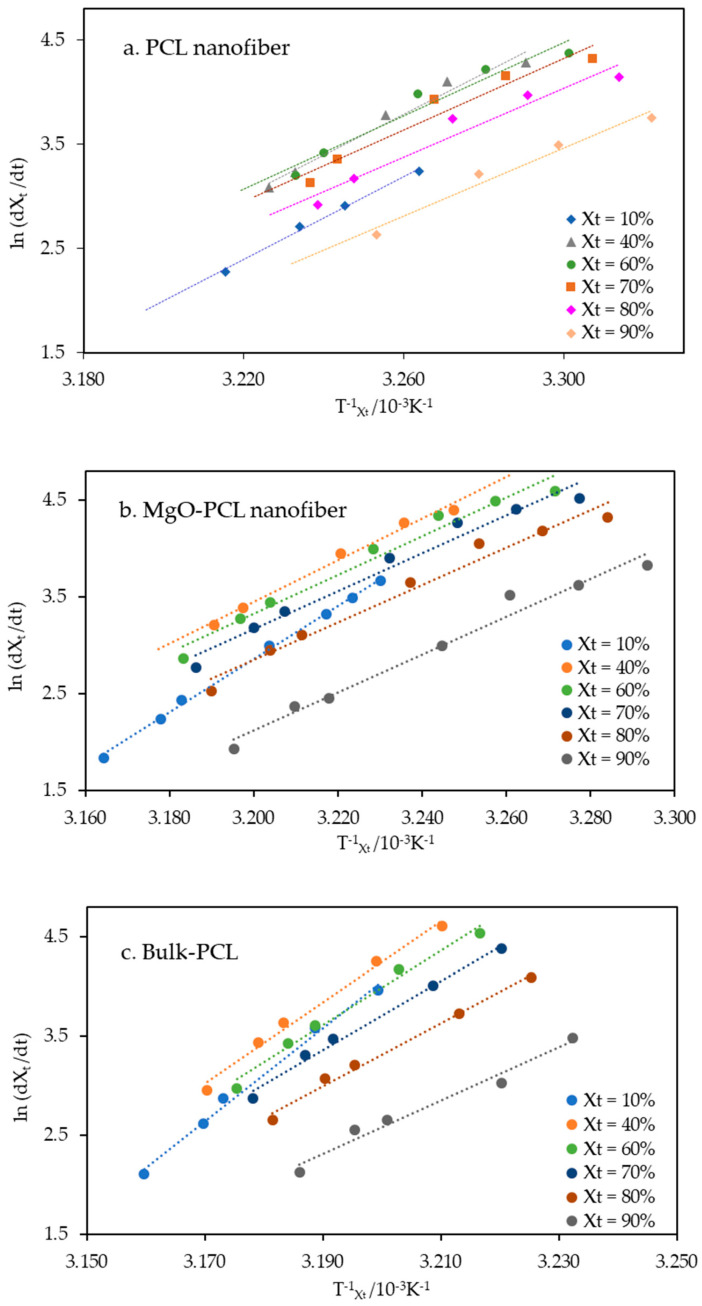
(**a**–**c**). Plots ln (*dX_t_*/*dt*) versus 1/*T_Xt_* of (**a**) PCL nanofiber, (**b**) MgO-PCL nanofibers and (**c**) Bulk-PCL at different relative crystallinities.

The activation energies of bulk-PCL, PCL, and MgO-PCL nanofibers are −364.74, −212 and −226.3 kJ/mol, respectively. The MgO-PCL nanofibers display a marginally reduced Δ*E* compared to the PCL nanofibers, with values of −226.3 kJ/mol and −212 kJ/mol, respectively. The present findings suggest that the incorporation of 5 weight percent magnesium oxide (MgO) into polycaprolactone (PCL) resulted in enhanced crystallization of PCL macromolecules as well as increased crystallization kinetics, owing to the nucleation potential of MgO. Moreover, it has been documented that there is an increase in activation energy (Ea) as the weight fractions of multi-walled carbon nanotubes (MWCNTs) increase within the range of 0.5 to 5% [42]. In 2002, Vyazovkin [51] demonstrated that the cooling processes are not subject to the Kissinger equation. It is suggested that the techniques of the differential iso-conversational approach, as proposed by Friedman in 1964, and the integral iso-conversional method, as suggested by Vyazovkin in 2001, are suitable for melt crystallization. The study will employ the Friedman methodology owing to its reliability and its uncomplicated nature. The equation known as the Friedman equation [52] can be mathematically represented as follows:

(13) $$\ln{\left(\frac{dX_{t}}{dt}\right)}_{X_{t}}=constant-\frac{\Delta E_{X_{t}}}{RT_{X_{t}}}$$

where *dX_t_*/*dt* represents the instantaneous crystallization rate concerning time given a specific value of relative crystallinity (*X_t_*), *R* is the universal gas constant (J mol^−1^ K^−1^), and Δ*E_X__t_* is the crystallization activation energy (kJ mol^−1^) that corresponds to crystallization temperature, *T_X__t_*, at various cooling rates. −Δ*E_X__t_*/*R* was determined from the slope coefficient plots of ln (*dX_t_*/*dt*) versus 1/*T_X__t_*, and exhibited a straight line, as shown in Figure 9a–c at each relative crystallinity. The high values of the regression coefficient (*R*^2^) provide evidence for the efficacy of the Friedman equation in the computation of activation energy for different degrees of crystal linearity.

**Figure 10:**
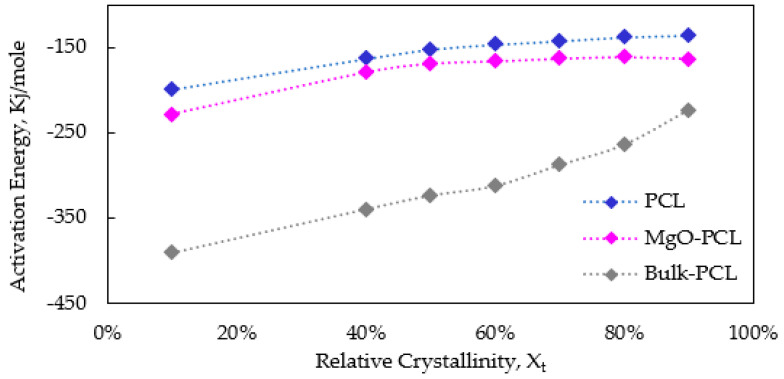
Activation energy dependence on the relative crystallinity for bulk-PCL, PCL, and PCL-MgO nanofibers.

The activation energy based upon Friedman equation on the reliance of the effective energy on the relative crystallinity is shown in Figure 10. The data indicates a positive correlation between the activation energy and the relative crystallinity. The data implies that the challenge of achieving crystallization in polymers intensifies with a rise in relative crystallinity. Activation energy for all samples follows the order: PCL nanofibers > MgO-PCL nanofibers > Bulk-PCL sample. Both nanofibers have a higher activation energy than Bulk-PCL, suggesting that crystallinity is reduced due to nanoconfinement of the nanofibers. As relative crystallinity increases, the temperature reduces, and activation energy increases. Therefore, the crystallization ability becomes more harder as the temperature reduces. Therefore, the higher the activation energy, the lower the crystallization ability and vice versa.

## 4. Conclusions

The non-isothermal crystallization kinetics of PCL and MgO-PCL nanofiber was assessed using calorimetry under different cooling rates. Mo’s method investigated the non-isothermal crystallization kinetics profile of bulk-PCL, PCL, and MgO-PCL nanofiber samples. The *α* value for these samples was found to be 1. The results suggest that the process of crystal nucleation and growth in all samples share a significant level of similarity, regardless of their levels of crystallinity. An increasing trend in the value of *F*(*T*) was observed as the relative crystallinity increases. It can be inferred that an increase in *F*(*T*) value results in increased crystallization difficulty as the relative crystallinity also rises. It was also observed that the crystallization process of MgO-PCL occurs at a higher rate than that of pristine PCL nanofibers at a specific relative crystallization rate.

Friedman’s approach determined the Ea values for bulk PCL, PCL nanofibers, and MgO-PCL nanofibers. The activation energy exhibits a negative value due to the dissipation of energy that occurs during the crystallization process from a molten state. The observed increase in activation energy (Ea) for nanofibers compared to bulk PCL suggests that confinement at the nanoscale hinders the transfer of PCL chain segments, thereby impeding the development of crystals.

## Figures and Tables

**Table 1:** Chemical compositions of bulk-PCL, PCL and MgO-PCL nanofibers.

Sample	Chemical Compositions		Voltage	Distance
	PCL (wt.%)	MgO (wt.%)	kV	cm
PCL	100	0	10	5
MgO-PCL	95	5	10	5
Bulk-PCL	100	0	-	-

**Table 2:** Crystallization behavior of bulk-PCL and confined PCL and MgO-PCL nanofibers.

Samples	Cooling Rate (K/min)	*T_o_* (°C)	*T_p_* (°C)	*t*_0.5_ (min)	Rate *t*^−1^_0.5_ (min^−1^)	Overall *t_c_* (min)	Crystallinity (%)
PCL	0.5	40.25	37.54	15.14	0.07	9.2	
	0.8	39.14	36.21	15.05	0.07	6.15	
	1	38.54	35.54	10.67	0.10	5.06	
	2	36.62	33.28	6.26	0.17	2.85	38
	3	35.47	31.75	4.46	0.23	2.46	
	4	34.28	29.84	2.94	0.26	1.97	
MgO-PCL	0.5	43.49	41.17	15.02	0.06	8.28	
	0.8	42.3	39.82	10.16	0.1	5.69	
	1	41.73	39.16	9.2	0.1	4.73	
	2	39.59	36.82	5.01	0.18	2.62	56
	3	38.5	35.3	4.05	0.25	1.99	
	4	37.58	34.01	2.53	0.22	1.66	
Bulk-PCL	0.5	43.93	42.00	7.89	0.13	7.74	
	0.8	43.07	41.16	4.76	0.21	4.83	
	1	42.69	40.75	3.87	0.26	3.94	
	2	41.2	39.31	2.5	0.40	2.14	60
	3	40.18	38.22	1.49	0.67	1.55	
	4	39.37	37.29	0.96	1.04	1.26	

**Table 3:** Parameters obtained from Jeziorny method for bulk-PCL, PCL, MgO-PCL nanofibers.

Primary Crystallization Stage					
Samples	ß (K/min)	*n*_1_	*Z_t_*	*Z_c_*	*R* ^2^
PCL	0.5	1.44	0.322	0.104	0.97
	0.8	1.20	0.875	0.846	0.97
	1	1.29	0.985	0.985	0.97
	2	1.38	0.691	0.831	0.97
	3	1.31	1.290	1.089	0.98
MgO/PCL	0.5	1.19	−0.156	0.487	0.97
	0.8	1.22	0.063	1.198	0.97
	1	1.20	0.179	1.512	0.97
	2	1.37	0.394	1.573	0.96
	3	1.22	0.762	1.795	0.97
Bulk/PCL	0.5	1.2	−0.176	0.444	0.98
	0.8	1.2	0.109	1.369	0.97
	1	1.3	0.216	1.644	0.97
	2	1.2	0.516	1.811	0.97
	3	1.3	0.794	1.840	0.97

**Table 4:** Non-isothermal crystallization kinetic parameters of bulk-PCL, PCL, and MgO-PCL nanofibers and obtained from Mo’s method.

Sample	*X_t_* (%)	20	40	50	60	80
Bulk PCL	*F*(*T*)	3.81	4.91	4.56	5.28	6.25
	*α*	1.12	1.11	1.11	1.11	1.12
MgO-PCL	*F*(*T*)	16.25	18.11	18.83	19.57	21.07
	*α*	1.33	1.32	1.32	1.31	1.30
PCL	*F*(*T*)	18.84	21.64	22.66	23.70	25.75
	*α*	1.475	1.451	1.441	1.433	1.415

**Table 5:** Activation energy values for bulk-PCL, PCL, MgO-PCL nanofibers.

Samples	Δ*E*/*R*	Δ*E* (kJ mol^−1^)
PCL	25.5	−212.01
MgO-PCL	27.05	−226.89
Bulk-PCL	43.87	−364.74

## Data Availability

The data presented in this study are available on request from the corresponding author.
